# Effect of Motor Imagery Training on Gait and Balance in Parkinson’s Disease

**DOI:** 10.7759/cureus.94710

**Published:** 2025-10-16

**Authors:** Srishtee Gautam, Rajendra Sharma, Shipra Chaudhary, Sakshi Jain, Kuljeet Anand

**Affiliations:** 1 Physical Medicine and Rehabilitation, Atal Bihari Vajpayee Institute of Medical Sciences and Dr. Ram Manohar Lohia (RML) Hospital, New Delhi, IND; 2 Physical Medicine and Rehabilitation, Employees' State Insurance Corporation (ESIC) Medical College and Hospital, Faridabad, IND; 3 Physical Medicine and Rehabilitation, Vardhman Mahavir Medical College and Safdarjung Hospital, New Delhi, IND; 4 Neurology, Atal Bihari Vajpayee Institute of Medical Sciences and Dr. Ram Manohar Lohia (RML) Hospital, Delhi, IND

**Keywords:** berg balance scale, motor imagery training, parkinson’s disease, six-minute walk test, unified parkinson’s disease rating scale

## Abstract

Objective: The aim of this study was to determine whether adding motor imagery training (MIT) to conventional exercise training (CET) improves gait and balance in patients with Parkinson’s disease (PD), compared with CET alone.

Methods: This prospective interventional study was conducted in the Department of Physical Medicine and Rehabilitation (PMR) in a tertiary care hospital from January 2021 to May 2022. Fifty patients with Stage 2-4 Hoehn and Yahr PD were included and divided equally into two groups. Group A received only CET, whereas group B received CET and MIT for 12 weeks. Assessment was done at baseline and at four, eight, and 12 weeks using the Berg Balance Scale (BBS), the Timed Up and Go test (TUG), and the Six-minute walk test (6MWT). Data were collected and analysed using various statistical tests by IBM SPSS Statistics software, version 21.0 (IBM Corp., Armonk, NY). A p-value < 0.05 was considered statistically significant.

Results: The two groups were comparable at baseline in terms of their demographic profile. Both groups had significant improvement (p<0.05) in all the assessment parameters at all follow-ups. A comparison between the groups revealed a better outcome in group B in terms of BBS and TUG, but the difference was not statistically significant, with p-values of 0.414 and 0.793, respectively, at 12 weeks. Both groups were comparable in terms of 6MWT at 12 weeks of follow-up.

Conclusions: Incorporating MIT into CET enhances outcomes related to gait and balance in patients with PD compared to CET alone.

## Introduction

Parkinson’s disease (PD) is a progressive, central nervous system (CNS) degenerative condition that typically affects elderly people [1]. The symptoms of PD include bradykinesia, stiffness, resting tremors, postural instability, and a variety of motor and non-motor symptoms [2]. Parkinson’s gait is characterized by anterior trunk flexion, reduction in step length, and decrease in walking speed [2]. Gait disturbances are associated with increased risk of falls at a later stage of disease, leading to morbidity and affecting overall quality of life.

Although pharmacological therapy is the mainstay of treatment of PD, physical exercise therapy minimizes or delays the evolution of symptoms and provides greater functionality, improving the overall quality of life [2]. Conventional exercise therapy (CET) mainly consists of range of motion exercises, strengthening exercises, exercises to improve endurance, training to improve balance and coordination, gait training, training for activities of daily living, etc.

Motor imagery training (MIT) is an emerging technique for the treatment of various neurological conditions. It activates the brain regions associated with movement, enabling previously unconscious actions to be accessed consciously [3]. In PD, MIT aims to improve motor function by training the brain to compensate for the deficits in movement execution. The mental practice in MIT includes visualization exercises where patients imagine themselves performing specific motor tasks repeatedly, strengthening the brain-muscle connection [2].

This study aims to determine whether adding MIT to CET improves gait and balance in patients with PD, compared with CET alone.

## Materials and methods

This was a prospective interventional study done in the Department of Physical Medicine and Rehabilitation in Atal Bihari Vajpayee Institute of Medical Sciences and Dr. Ram Manohar Lohia (RML) Hospital, a tertiary care hospital in New Delhi, India, from 1^st^ January 2021 to 31^st^ May 2022. Prior institutional ethical clearance was granted from the Institutional Ethical Committee with IRB number TP(MD/MS)(106/2020)/IEC/ABVIMS/RMLH/37.

Patients with a confirmed diagnosis of PD, classified within Hoehn and Yahr [4] Stages 2 to 4, without cognitive impairment, and with the ability to perform motor imagery in the kinaesthetic modality were included in the study. Patients were excluded if they had a history of hypertension, were receiving psychotropic medications, had undergone deep-brain stimulation surgery, or presented with any comorbid conditions that could impair gait, vision, or hearing.

Based on a previous study by da Silva et al. [2], the sample size came out to be 40 in each group using the formula:

$N = \frac{(s_1^2 + s_2^2) \left[ \left( z_{1-\alpha} + z_{1-\beta} \right)^2 \right]}{(\chi_1 - \chi_2)^2}$

where χ1 is the mean of group 1, χ2 is the mean of group 2, s1 is the standard deviation of group 1, s2 is the standard deviation of group 2, z(1-α) is the value of the normal deviate at the considered level of confidence for a one-tailed test, and z(1-β) is the normal deviate at the considered power of the study.

However, since this study was undertaken during the COVID-19 pandemic, the sample size was reduced to 25 for each of the two groups owing to significantly reduced patient mobilization at the hospital, given the emergency protocols in place.

Fifty patients were recruited and divided into two groups, Group A and Group B, in an alternating manner. Group A was given a CET (Appendix 1) for 45 minutes, three days a week, whereas Group B was given a CET for 45 minutes along with MIT for 20 minutes, also three days a week. Exercises were taught to the patients, and they were advised to do them at home. Compliance was ensured through tele-rehabilitation and video clip sharing. Patients had regular follow-up visits and were assessed at baseline, four weeks, eight weeks, and 12 weeks with the following assessment tools: Six-Minute Walk Test (6MWT) [5], Berg Balance Scale (BBS) [6], and Timed Up and Go Test (TUG) [7].

Data were entered in Microsoft Excel (Microsoft Corporation, Redmond, WA) and analysed using IBM SPSS Statistics software, version 21.0 (IBM Corp., Armonk, NY). Categorical variables were expressed as counts and percentages, while continuous variables were reported as mean ± SD or median (IQR), based on data distribution. Associations between categorical variables were analysed using the chi-square or Fisher’s exact test. For between-group comparisons, the independent t-test or Mann-Whitney U test was applied. A p-value < 0.05 was considered statistically significant.

## Results

The mean age in group A was 63.72 ± 9.4 years, while in group B it was 62.20 ± 9.11 years. Out of a total of 50 patients recruited, there were a total of 18 females and 32 males. In both groups, males outnumbered females. In group A, the majority of patients (40%) were of Hoehn and Yahr Stage 2.5. In group B, 52% of patients were in Stage 2.5. Both groups were comparable in terms of their demographic profile and staging (Table 1).

**Table 1:** Demographic profile and Hoehn and Yahr stage in both the groups

Characteristics		Group A	Group B	p-value	Statistical values
Age (years, mean ± SD)		63.72±9.40	62.20±9.11	0.564	independent t statistic – 0.581
Gender (female:male)		11:14	7:18	0.239	Chi-square=1.389
Hoehn and Yahr Stage	Stage 2	6 (24%)	8 (32%)	0.381	Fisher-Freeman-Halton exact test = 2.915
	Stage 2.5	10 (40%)	13 (52%)		
	Stage 3	8 (32%)	4(16%)		
	Stage 4	1 (4%)	0		

On assessment at BBS, significant improvement in both groups at the eight-week and 12-week follow-ups was observed when compared to baseline. On intergroup comparison, the median values of BBS showed better improvement in group B than in group A. Values were not statistically significant, as shown in Table 2.

**Table 2:** Comparison of Berg Balance Scale (BBS) score at various follow ups of both the groups *Dunn’s pairwise post-hoc analysis

	Group A			Group B			p-value (Intergroup comparison)	Mann-Whitney U test value
	Median (IQR)		p-value (comparison from baseline)	Median (IQR)	p-value (comparison from baseline)			
Baseline	44 (34.5-50)			44.5 (42-49.5)			0.100	U = 397.0
4 weeks	44 (39-50)		0.566*	45.5 (44-50)	0.397*		0.059	U=409.5
8 weeks	46 (40.5-50.5)		0.006*	50 (44-52)	0.029*		0.054	U=310.0
12 weeks	48 (44-54)		<0.001*	50.5 (47.25- 54)	<0.001*		0.414	U=241.0
Friedman's two-way analysis of variance	p-value <0.001	F value 35.72		p-value <0.001		F value 26.93		

Similar findings were observed in the TUG test. The TUG test decreased significantly in both groups at various follow-ups, and in group A, the median time decreased from 22 seconds at baseline to 18 seconds at 12 weeks. Whereas in group B, this improvement was more as time decreased from 22 seconds to 15 seconds at 12 weeks. However, on comparison, the p-value was not statistically significant between the groups at any of the follow-ups, as shown in Table 3.

**Table 3:** Timed Up and Go (TUG) score of both the groups at various follow ups *Dunn’s pairwise post-hoc analysis

	Group A			Group B			p-value (Intergroup comparison)	Mann-Whitney U test value
	Median (IQR)	p-value (comparison from baseline)		Median (IQR)	p-value (comparison from baseline)			
Baseline	22 (13.75- 37)			22 (18.25-30)			0.683	U = 291.5
4 weeks	21 (12-35)	0.02*		19.5 (16.25- 28.5)	0.120*		0.496	U=277.5
8 weeks	21 (12-35.5)	<0.001*		16 (14.25- 26.75)	<0.001*		0.496	U=203.0
12 weeks	18 (11.5- 39)	<0.001*		15 (12-27.58)	<0.001*		0.793	U=200.0
Friedman's two-way analysis of variance	p-value <0.001		F value 41.74	p-value <0.001		F value 40.60		

The 6MWT distance increased significantly at eight and 12 weeks in both groups when compared to their respective baseline values. The improvement in 6MWT was comparable in both groups (p > 0.05), as shown in Table 4.

**Table 4:** Six-Minute Walk Test (6MWT) score of both the groups at various follow ups *Dunn’s pairwise post-hoc analysis

	Group A			Group B			p-value (Intergroup comparison)	Mann-Whitney U test value
	Median (IQR)	p-value (comparison from baseline)		Median (IQR)	p-value (comparison from baseline)			
Baseline	200 (103.5- 251)			168.5 (123.75-194.25)			0.877	U = 320.5
4 weeks	216 (136-266.5)	0.292*		178.5 (140- 203)	0.086*		0.547	U=281.5
8 weeks	216 (115.5-284)	0.001*		183 (151- 212)	<0.001*		0.865	U=238.0
12 weeks	230 (171-288)	<0.001*		196 (180-246.75)	<0.001*		0.223	U=256.5
Friedman's two-way analysis of variance	p-value <0.001		F value 44.243	p-value <0.001		F value 40.337		

## Discussion

MIT is an emerging approach in neurological rehabilitation. This study aimed to evaluate whether combining MIT with CET offers greater benefits in managing PD symptoms than CET alone. Outcomes were assessed using 6MWT and TUG for gait and BBS for balance.

Results in the present study demonstrated significant improvements in both groups following 12 weeks of intervention. CET alone showed notable benefits in gait and balance, aligning with previous studies [8-11]. Participants receiving both MIT and CET also experienced significant within-group improvements, corroborating results from earlier investigations [12-14].

However, while the group receiving combined MIT and CET showed better outcomes across gait and balance than the CET-only group, these differences were not statistically significant. This is consistent with findings from prior studies by Braun et al. [15] and da Silva et al. [2], which also found no significant advantage of MIT over conventional therapy in similar PD cohorts.

Some studies have reported MIT as superior to conventional therapy [16-19], though variations in study design may explain discrepancies. First, this study included a larger sample size (n=50) compared to 23-26 in earlier studies [16-18]. Second, patients with Hoehn and Yahr Stages 2-4 were included in the present study, while other studies focused on milder cases (Hoehn and Yahr Stages 1-3) [16,17,19]. Given the difficulty PD patients experience initiating movement, motor imagery may be more effective in early stages than in moderate to severe cases [20].

Lastly, different studies have used variable outcome measures. Therefore, it is very difficult to compare the findings of previous studies with our study. In this study, outcomes were assessed in terms of gait and balance by using TUG, 6MWT, and BBS. In this study, there was no significant difference between group analyses in any of these parameters. In one of the randomized control trials, they used only the Unified Parkinson's Disease Rating Scale (UPDRS) III and compared MT to conventional therapy. Their findings were in favor of the MIT group only in three parameters of the UPDRS III scale [20]. In another study by Tamir et al. on the effectiveness of MIT, the outcome measures TUG and UPDRS II, III, and VI were used. They observed significant improvement in the MIT group in TUG [16]. Similarly, in a previous study by Sarasso et al., in addition to other outcome parameters, TUG and UPDRS III were used. They have found comparable results of motor imagery with exercise training in patients with PD, which were in accordance with our results [18]. These methodological differences likely account for the variability in reported outcomes.

Limitations

This study has several limitations. First, the sample size was relatively small, and only patients with Hoehn and Yahr Stages 2-4 were included without randomization and blinding. Second, comorbidities were not considered in the outcome analysis, potentially influencing the results. Third, the follow-up duration was limited to 12 weeks, which may not fully capture the long-term effects of MIT.

## Conclusions

MIT appears to be a promising and beneficial adjunct in the rehabilitation of individuals with PD. Although the integration of motor imagery with conventional exercise programs led to observable improvements in balance and gait, the differences observed did not reach statistical significance in this study. Nonetheless, the clinical relevance of these improvements should not be overlooked, particularly given the progressive nature of PD and the need for multimodal rehabilitation strategies that are both effective and accessible.

The findings suggest that motor imagery may enhance neural plasticity and motor learning in individuals with PD, supporting its theoretical foundation in neurorehabilitation. However, the lack of statistically significant differences underscores the need for further investigation. Future research should aim to address the limitations of the current study, including a relatively small sample size, short intervention duration, and lack of blinding. Well-designed randomized controlled trials with larger cohorts, longer follow-up periods, and rigorous methodology are essential to confirm the efficacy of motor imagery therapy and to establish standardized protocols for its implementation in clinical practice.

## Appendices

Appendix A

Conventional Exercise Training Program

**Table 5:** Conventional exercise training program

Week-wise program
Week 1
Flexibility exercises (5 repetitions, 15-second hold)
Hamstring stretch
Gluteus maximus and hip flexor stretch
Gastrocnemius and soleus stretch
Paraspinal stretch
Strengthening exercises (baseline determination of preferred elastic band strengths for lower
Limb exercises- 1 repetition maximum)
Lower limb muscles(elastic band: 1 set of 8-10 reps for each leg)
Quadriceps (sitting and straight leg raises)
Hamstrings
Gluteus maximus
Gluteus medius
Upper limb muscles (5-10 reps)
Push-ups
Abdominal muscles (5 reps)
Curl-ups with arms behind head
Instruction in body mechanics for:
- Standing
- Sitting
- Lying
- Lifting
- Reaching
- Carrying
- Arising from the floor
- Ascending/descending stairs
Baseline walking evaluation (determine maximum comfortable distance)
Week 2
Flexibility exercises (as above)
Strengthening exercises: lower limb muscles (elastic band: 1 set of 10 reps, each leg), upper
limb muscles(10 reps), abdominal muscles (5- 10 reps )
Postural exercises (10reps, 10 sec hold)
Head and neck
Trunk
Coordination exercises
Reciprocal leg movements (10 reps, eyes closed)
Bridging (10 reps)
Sitting/ standing (5 reps)
Braiding exercises (2 reps)
Reciprocal ankle motion (10 reps)
Rung ladder: forward stepping (2 reps)
‘Survival’ maneuvers
Floor recovery exercises- “how to get up if you should fall “
Ascending and descending stairs safely (individual practice)
Endurance walking (begin at 75%- 100 % of baseline minutes walked; increase at a comfortable pace)
Week 3
Flexibility exercises (5 reps, 20 sec hold)
Strengthening exercises: lower limb (2 sets of 10 reps), upper limb (push-ups; 10-15 reps), abdominals (curl-ups, 10-15 reps)
Postural exercises (15 reps, 10 sec hold)
Coordination exercise(repetitions increased)
Survival maneuvers
Endurance walking (0-6 minutes, comfortable speed)
Week 4
Flexibility exercises(5 reps, 25 sec hold)
Strengthening exercises: lower limb (2 sets of 10 reps), upper limb (push-ups; 10-15 reps), abdominals (curl-ups, 10-15 reps)
Postural exercises (20 reps, 10 sec hold)
Coordination exercise (repetitions increased)
Reciprocal legs (eyes closed )
Braiding (no holding, eyes open)
Rung ladder (forward, side, and backward stepping)
Survival maneuvers
Endurance walking (3-8 minutes, comfortable pace)
Week 5
Flexibility exercises(5 reps, 30 sec hold)
Strengthening exercises: lower limb (3 sets of 10 reps), upper limb ( push-ups, 15-20 reps), abdominals (curl-ups, 15-20 reps)
Postural exercises(25reps, 10 sec hold)
Coordination exercise(repetitions increased)
Reciprocal ankle dorsi/ plantar flexion (25 reps )
Braiding (no holding, eyes open)
Survival maneuvers
Endurance walking( 6-10 minutes, comfortable pace)
Week 6
Flexibility exercises (5 reps, 30 sec hold)
Strengthening exercises: lower limb (3 sets of 10 reps), upper limb (push-ups, 20 reps), abdominals (curl-ups, 15-20 reps)
Postural exercises (25 reps, 10 sec hold)
Coordination exercise (repetitions increased)
Survival maneuvers
Endurance walking (8-12 minutes, comfortable pace)
